# Urologic Manifestations and Hydronephrosis as Initial Presentation of Erdheim-Chester Disease: A Rare Form of Non-Langerhans Histiocytosis

**DOI:** 10.7759/cureus.19750

**Published:** 2021-11-19

**Authors:** Skyler E Burke, Akriti Chaudhry, Erin A Kaya, Kyle C Schuppe, Cheddi Thomas, Shane M Pearce, Henry Mroch

**Affiliations:** 1 Oncology, Washington State University Elson S. Floyd College of Medicine, Spokane, USA; 2 Hematology and Medical Oncology, Summit Cancer Centers American Oncology Partners, Spokane, USA; 3 Medicine, Washington State University Elson S. Floyd College of Medicine, Spokane, USA; 4 Pathology, Washington State University Elson S. Floyd College of Medicine, Spokane, USA; 5 Urology, Spokane Urology, Spokane, USA; 6 Nephrology, Washington State University Elson S. Floyd College of Medicine, Spokane, USA

**Keywords:** pericardial infiltration, non-langerhans histiocytosis, erdheim-chester disease, retroperitoneal fibrosis, hydronephrosis

## Abstract

Erdheim-Chester disease (ECD) is a rare non-Langerhans histiocytosis that is classified as a malignancy of myeloid progenitor cells, with only 1,000 confirmed cases in the literature so far. It often manifests as a multi-system disorder with an initial presentation predominantly in the long bones, central nervous system (CNS), and retroperitoneal space, sometimes causing urologic symptoms as a result. ECD often presents indolently and in a spectrum of different ways, making it challenging to identify and treat. We report a case of a 63-year-old female with ECD that first presented with abdominal pain and acute renal injury due to ECD-related retroperitoneal fibrosis. We also explore the literature at large around ECD, its diagnosis, pathophysiology, and advances in treatments.

## Introduction

Erdheim-Chester disease (ECD) is a rare subtype of non-Langerhans histiocytosis associated with the proliferation of myeloid-derived non-Langerhans cell histiocytes. Since it was first described in 1930 by Jakob Erdheim and William Chester, there have only been approximately 1,000 confirmed cases reported in the literature [1,2]. The pathology of ECD involves the deposition of proliferating macrophages that eventually become lipid-laden xanthomas. These cells then form inflammatory necrotic xanthogranulomatous lesions with fibrotic deposition leading to many of the clinical presentations most commonly seen in ECD [3]. A retrospective study found that ECD most commonly affects the bone, specifically the femur, tibia, and fibula, followed by the retroperitoneum and large vasculature, and finally the heart, which is the main cause of mortality in ECD [4,5].

In this report, we describe a rare case of a patient diagnosed with ECD based on the presentation of urologic manifestations and subsequent imaging that showed retroperitoneal fibrosis. Further studies are needed on this rare clinical disease in order to better understand and optimize diagnosis and treatment for ECD patients in the future.

## Case presentation

**Figure 1:**
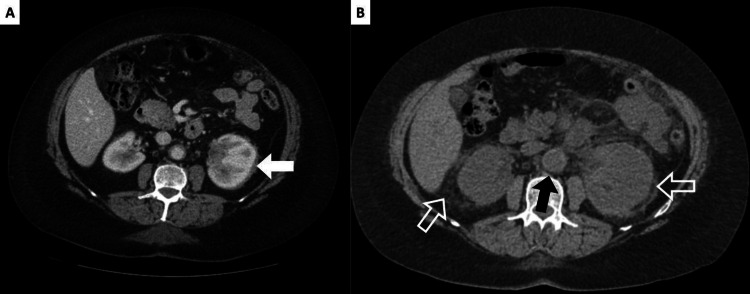
Axial CT scan of the abdomen (A) Initial axial CT scan of the abdomen demonstrating perinephric stranding with "hairy kidney" features (solid white arrow). (B) Axial CT scan of the abdomen at the one-year follow-up demonstrating periaortic thickening (solid black arrow) and expanded bilateral perinephric stranding and thickening (hollow white arrows) CT: computed tomography

A 63-year-old female presented with acute left lower quadrant pain and a one-month history of inguinal numbness. A CT of the abdomen and pelvis with contrast indicated bilateral perirenal thickening greater on the left kidney and stranding nodularity. Thickening and nodular infiltrate were also present in the peritoneum as well as mild stranding around the aorta and iliac arteries bilaterally (Figure 1A).

Based on the finding of retroperitoneal fibrosis, the patient was referred to nephrology. At that time, her serum creatinine was 1.3 mg/dl with the rest of her physical examination unremarkable. Her symptoms were initially well controlled with naproxen, prednisone, and tamoxifen, but later the tamoxifen was discontinued due to side effects, namely chest pain.

One year later, the patient presented with right flank pain and anemia. A CT scan showed increased bilateral renal fat stranding and perinephric soft tissue thickening, on the left kidney more than the right, when compared to the prior CT. Her kidneys were edematous and extending into the renal pelvis, again with the left greater than the right. In the peritoneum and retroperitoneum, extensively increased soft tissue stranding was seen along the anterior peritoneal surface lateral to the ascending and descending colon. Additionally, both adrenal glands appeared thickened and edematous bilaterally, which was a new finding. Similar to the prior CT scan, soft tissue thickening was seen on the abdominal aorta and proximal bilateral common iliac vessels as well as the superior mesenteric artery extending 5 cm from its origin (Figure 1B).

Following this imaging, the patient was referred to urology. She presented with intermittent bilateral flank pain, abdominal pain, nausea, decreased appetite, and a 20-pound weight loss over several weeks. Diagnosed with retroperitoneal fibrosis and an acute kidney injury (serum creatinine of 1.81 mg/dl), urology placed bilateral ureteral stents and recommended a possible future definitive staged bilateral ureterolysis.

**Figure 2:**
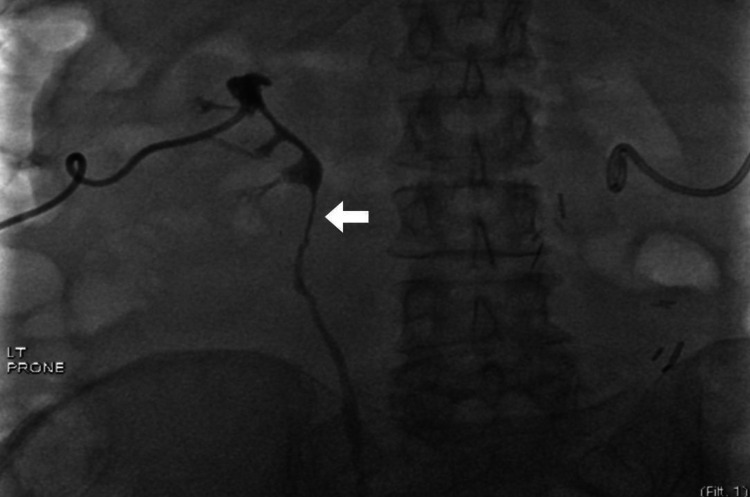
Antegrade pyelogram Antegrade pyelogram with ureteral tapering (solid white arrow)

After a few weeks of respite, the patient's symptoms progressed to the point of requiring hospital admission. Upon evaluation, she was septic and had acute on chronic kidney disease stage 3 with a glomerular filtration rate (GFR) of 13 mL/min and serum creatinine had increased to 4 mg/dl. Ultrasound and abdominal plain film demonstrated proper placement of the stents. Retrograde pyelograms demonstrated bilateral proximal ureteral obstruction with extrinsic compression of the proximal ureter and renal pelvis (Figure 2).

**Figure 3:**
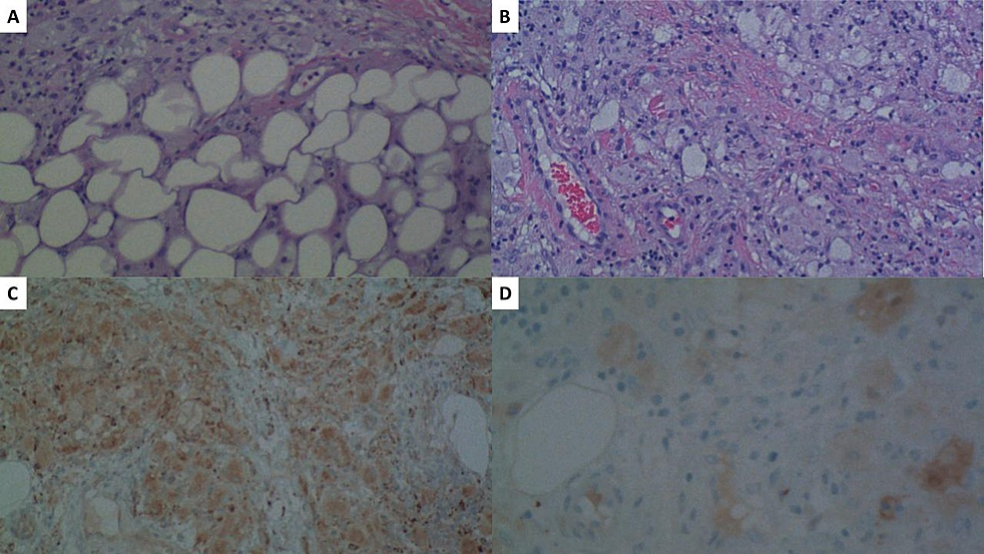
Immunostaining of the pericardium tissue (A), (B) Dense collections of histiocytes with foamy cytoplasm in a fibrotic background. (C) CD68 immunostain at 100x magnification is positive within tumor cells, confirming histiocytic differentiation. (D) S100 immunostain at 200x magnification is also positive within tumor cells

Four months later, the patient had a pericardial effusion with near tamponade. The tissue pathology from her pericardial window surgery showed diffuse pericardial infiltration with histiocytic infiltrate expressing CD68 and a subset expressing the S100 protein. The pre-immunostain differential included a variety of diagnoses including metastatic carcinoma, histiocytosis, and, less likely, melanoma. The markers identified were highly associated with ECD, and this was the first time that ECD was suspected (Figure 3).

**Figure 4:**
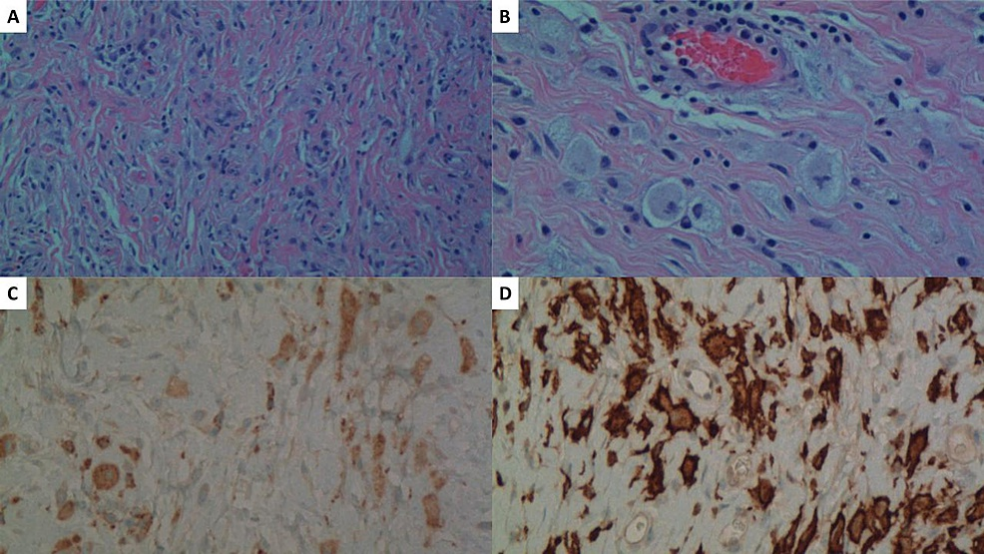
Immunostaining of omental tissue (A), (B) H&E stain at 100x magnification (A) and 200x (B) showing a dense collection of histiocytes with foamy cytoplasm in a fibrotic background. (C) Immunostain for CD68 at 200x magnification is positive within tumor cells, confirming histiocytic differentiation. (D) Immunostain for CD163 at 200x magnification is also positive within tumor cells, further confirming histiocytic differentiation

One month after this biopsy report, the patient underwent an exploratory laparotomy, omental biopsy, and right ureterolysis. At that time, she had bilateral nephrostomy tubes placed due to ureteral obstruction. She was counseled on the possibility of a bilateral ileal ureteral substitution with attendant risks. Upon surgical exploration, she was found to have dense fibrosis involving large and small bowel mesentery, omentum, serosa, and retroperitoneum, predominantly at the level of the right renal pelvis and hilum. The retroperitoneal fibrotic process was encasing the entire right kidney. The fibrotic process was extremely dense with a "woody" consistency and continued up to the renal hilum. Omental biopsy of representative tissue consistent with the retroperitoneal process was taken and sent for a second opinion to several groups. Final histopathology showed non-Langerhans histiocytosis, consistent with ECD (Figure 4).

At that time, the patient was referred to a multidisciplinary tumor board for the best course of further diagnostic testing and treatment. Upon genetic testing, the patient was *MAP2K1-*positive while *KRAS-* and *NRAS-*negative. She was negative for *BRAF^V600E^*, and hence a titrated dose of cabozantinib, a small molecule inhibitor of the tyrosine kinases c-Met and VEGFR2, AXL, and RET was used instead of vemurafenib (an inhibitor of the mutated *BRAF^V600E^* kinase). She was started on cabozantinib 20 mg daily, which was increased to 40 mg a week later, and then increased again to 60 mg the following week and maintained at that daily dose. Alternative tyrosine kinase inhibitors may be considered in the future if her ECD is refractory.

## Discussion

ECD usually presents in adulthood with an average age of onset in the mid-50s, and it is rarely diagnosed in children. Both men and women are affected, but men account for more of the reported cases at a ratio of 3:1 [2]. In this case report, we discussed the diagnosis of ECD in a 63-year-old female.

Currently, no clear hereditary association related to ECD has been identified. The development of ECD has not yet been correlated or associated with any known environmental risk factors. As ECD is a hematologic malignancy, it logically follows that any behavior that is known to induce genetic mutations like drinking, smoking, and exposure to ionizing hazardous materials could cause a somatic mutation leading to ECD, but this connection has not yet been established [6,7]. Our patient did have a six-year history of smoking tobacco; it is not clear if there is an association between that and her eventual development of ECD.

ECD is caused by an overexpression of myeloid progenitor cells. One contributory gene is *BRAF,* which encodes the transduction protein kinase B-Raf and is a component of the MAP kinase signaling pathway and enables the proliferation and expansion of myeloid progenitor cells [8]. In particular, the *BRAF^V600E^* mutation is strongly correlated with ECD. In over half of the patients with ECD, genetic testing will indicate the presence of *BRAF^V600E^* and in fact, this number may be even higher if sensitive enough genetic testing techniques are used [9]. One explanation for the varied phenotype and severity of ECD seen clinically could be related to clonal expansion of myeloid progenitors each with a varied immunological and physiological functional profile. Further complicating this consideration is the fact that a spectrum of inflammatory cytokines and chemokines are produced by the malignant histiocytes, which can lead to more recruitment of inflammatory cells and consequent damage to healthy tissue. Arnaud et al. found that IL-6, IFN-ɑ, and MCP-1 levels, in particular, were elevated in ECD patients while contrarily, IL-4 and IL-7 were decreased. This suggests that the malignant histiocytes activate Th-1 lymphocytes and thus macrophages and eosinophils, further perpetuating a fibrotic and autoimmune-predominant effect in impacted tissues [10].

ECD is challenging to identify due to a lack of clear pathognomonic presentation. Even imaging can be non-specific, and hence a biopsy suggestive of ECD followed by genetic testing is essential for the diagnosis. However, on reviewing the literature, it is clear that certain clinical presentations predominate. One review of 259 patients with confirmed ECD found that the most common initial presentation is dull bone pain (26%), neurologic findings (23%), diabetes insipidus (22%), and constitutional symptoms (20%) [2]. The most common imaging finding is radiographic evidence of diaphyseal and metaphyseal osteosclerosis in the legs. The gold standard for visualization of these lesions is a bone scan, but they can also be characterized well via PET or less sensitively by CT/MRI. These lesions are not seen on an X-ray. While disease sites in the long bones were seen in 95% of patients, Arnaud et al. found that in a retrospective analysis of imaging of 37 patients with ECD, the large vessels, retroperitoneum (59% each), and heart (57%) were also very commonly involved [5]. Hence, the recommended modality for initial assessment includes CT of the chest, abdomen, and pelvis followed by an (18F)-fluorodeoxyglucose (FDG) PET scan of the entire body in place of a dedicated bone scan. In advanced cases, more detailed studies like MRIs of the brain and heart may be recommended to assess central nervous system (CNS) involvement, which is associated with a poor prognosis [9].

One study found that in 4% of patients with ECD, osteosclerosis is not seen on imaging or not present at all, and this was true for the patient we described in this report [10]. Our patient had an initial complaint of abdominal pain and acute on chronic kidney disease. This was due to her progressive retroperitoneal fibrosis and perinephric fat stranding or infiltrate. This finding, dubbed "hairy kidney" is a common incidental finding on CT, and the associated retroperitoneal fibrosis is seen in 30% of patients [9-11]. The retroperitoneal fibrosis seen in ECD patients like the one described here can be differentiated from idiopathic retroperitoneal fibrosis as, in the latter, the ureters in the pelvis and the IVC are usually also fibrotic. ECD-associated retroperitoneal fibrosis can constrict the ureters and renal pelvis causing hydronephrosis and requiring stenting or nephrostomy, which was the case with our patient [9]. In fact, one institutional analysis of 47 patients found that 37 patients (79%) had evidence of ECD-related urological involvement. This included 28 with retroperitoneal infiltration (60%) like in our patient, 21 with lower urinary tract symptoms related to diabetes insipidus (DI) (45%), 10 with hydronephrosis (21%), and 18 with chronic kidney disease (38%) [12]. All of these were present in our patient besides a defined diagnosis of DI. While it is unlikely that our patient had central DI due to no CNS involvement, it can be difficult to distinguish the two with initial imaging due to the simultaneous CNS and renal involvement of ECD. Interestingly, the development of DI in patients with ECD can actually occur instead due to CNS ECD infiltration of the pituitary gland disrupting normal endocrinology [13].

Fibrotic deposition around the abdominal aorta and branches like the iliac arteries is also often seen and is noted to be a "coated aorta" in such cases [11,14]. The ECD deposition around vessels can be an asymptomatic finding and is often incidentally discovered via imaging, as was the case with our patient [8]. However, as in the case with our patient, this cardiovascular involvement can progress to pericardial effusion and even cardiac tamponade. This is relatively common as pericardial complications are present in 40-45% of patients and cardiovascular events are a major cause of death in ECD patients [9,11]. The presentation of ECD is non-specific but should be suspected in all cases of bilateral long bone pain accompanied by CNS abnormalities like gait instability, neuropathy/numbness, and fatigue as well as progressive renal pathologies with fibrotic deposition on imaging.

Among patients with ECD, disease prognosis is generally variable and multifactorial. In a retrospective study of 165 ECD patients, the overall mortality was 24.8% and median survival was 162 months with a five-year survival of 82.7%. The main presentations that resulted in worse outcomes and survival were CNS, retroperitoneal, and lung involvement. Cardiac and CNS involvement is often associated with the *BRAF^V600E^* mutation but the mutation alone was not an independent predictor of survival [15]. In another study of 44 patients, the authors found that patients who were older than 60 years with digestive organ involvement had worse outcomes and that, in general, CNS and cardiovascular involvement was also associated with diagnosis at an older age [16]. This is an important consideration for our patient as her older age and cardiovascular and digestive organ involvement could be risk factors for progressive disease and future CNS involvement.

Treatment for ECD poses many challenges as it is a rare disease that can present in many different ways, often indolently. Immune system modulators and targeted therapies have been shown to be helpful for reducing the local inflammatory environment for ECD patients. The innate immune system stimulator pegylated interferon-α is associated with improved survival in ECD patients and is recommended as the first-line treatment for patients with bone, skin, renal, or aortic coating, which was the case with our patient [11,17]. It is theorized that IFN-α is beneficial for ECD patients as it functions to inhibit the actions of IL-1 and for that reason, anakinra (an IL-1 inhibitor) has shown some promise in a small patient trial. Interestingly, in one patient with ECD-related cardiac disease similar to our patient, anakinra was shown to be successful [18]. Progress has been made in the field of immunologics with relatively recent developments of inhibitors of the mutated *BRAF^V600E^* kinase like vemurafenib. In three patients treated with vemurafenib, a substantial response was noted, including a confirmed reduction in tumor burden via PET scan [19]. In a more recent clinical trial of 18 patients with histiocytic neoplasms, the administration of an oral inhibitor of MEK1 and MEK2 called cobimetinib resulted in an 89% response rate even in patients who were *BRAF^V600E^-*negative [20]. While one study estimates that almost half of ECD patients test positive for the *BRAF^V600E^* mutation,* *less than a quarter test positive for *MAP2K1* mutations like the patient described here. In such instances, a consensus recommendation is to start the patient on a tyrosine kinase inhibitor like cabozantinib, which was the treatment modality used for our patient [21].

## Conclusions

In this case report, we discussed the presentation and diagnosis of a patient with ECD with renal and cardiovascular manifestations. ECD is a rare and challenging illness to diagnose and treat. The spectrum of symptom severity ranges widely from indolent to rapid progression of the disease, which can be deadly. Our patient’s initial complaint of abdominal pain and numbness was vague, but imaging of the abdomen can reveal early stages of fibrotic deposition of ECD. A biopsy combined with classic clinical features may strongly suggest ECD but a genetic test for the *BRAF^V600E^* mutation would be almost definitive and also provide evidence of a targetable condition with kinase inhibitors like vemurafenib. Considering the possible multisystem involvement in ECD, especially in advanced conditions, it is critical to establish a multidisciplinary care team. With the cardiovascular, renal, and CNS manifestations of ECD, early detection, coordination, and treatment are critical. Utilization of novel *BRAF^V600E^* inhibitors like vemurafenib or additional tyrosine kinase inhibitors in aggressive or refractory cases of ECD could prevent disease sites in life-threatening regions from progressing to an acute state, thereby preventing stays in ICUs or death. Further studies are required to gain deeper insights into the diagnosis and treatment of this rare malignancy.
